# Having Difficult Conversations: The Advanced Practitioner’s Role

**Published:** 2013-01-01

**Authors:** Therese Svarovsky

**Affiliations:** From Seidman Cancer Center, Cleveland, Ohio

## Abstract

Throughout the course of their disease, patients with cancer and their families look to the oncology advanced practitioner (AP) for support and guidance as they struggle with the emotional impact of a life-limiting illness, complex treatment decisions, and the challenge of sustaining hope while maintaining realistic goals. At various points along the disease trajectory, difficult conversations between the AP and the patient are essential. In this case report of a 43-year-old woman with metastatic breast cancer, we model the use of various strategies available for the AP to help make these difficult conversations as productive, relevant, and emotionally safe for the patient as possible.

Patients with cancer report unmet needs for communication of information about the extent of their disease, prognosis, treatment options, intent, and adverse effects (Kissane et al., 2012). The oncology advanced practitioner (AP) needs to be equipped to answer patients’ questions in an honest and empathic manner, to consider the varied informational and emotional needs of their patients, and to provide patient-centered recommendations for future treatments (Saraiya, Arnold, & Tulsky, 2010).

Throughout the disease trajectory, patients and their families look to the AP for support and guidance. Patients with advanced and incurable cancers struggle with the emotional impact of a life-limiting illness, complex treatment decisions (often involving clinical trials), and the challenge of sustaining hope while maintaining realistic goals (Back et al., 2008).

Over the past century, developments in public heath and new medical therapies have led to an exponential increase in the longevity of cancer patients (Goldstein, Back, & Morrison, 2008). Because patients are living longer than ever before, the communication challenges faced by oncology clinicians have become ever more complex, involving uncertainty, hope, and even discussion of widely available anecdotes of patients who "beat the odds" (Back et al., 2008).

## Managing Difficult Conversations

A difficult conversation can be defined as one that takes place between the AP and the patient at transition points on the disease trajectory. Clinicians and researchers widely acknowledge the importance of addressing not only physical but also emotional and spiritual suffering at the end of life (Steinhauser et al., 2008). A well-conducted difficult conversation can aid in establishing the multifaceted goals of care to include the physical, spiritual, and psychosocial domains. It also provides a forum for setting realistic goals and sustaining hope.

Goals of care are defined and reevaluated throughout the disease trajectory. When an AP raises the possibility of discontinuing chemotherapy for a patient with advanced cancer, a turning point occurs between the AP and the patient (Back et al., 2010)—one that most often necessitates a difficult conversation. This turning point or transition conversation can be a part of an introduction to a larger discussion about what the patient wants to accomplish or avoid at the end of life (Back, Bauer-Wu, Rushton, & Halifax, 2009). The conversation is best conducted when a therapeutic relationship based on trust and mutual respect has already been established (Back et al., 2008).

## Training for Difficult Conversations

Unfortunately, the communication skills required for an effective difficult conversation go beyond the basic interviewing techniques that are taught in health-care professional training schools. Some institutions are participating in the End-of-Life Nursing Education Consortium (ELNEC), administered by the American Association of Colleges of Nursing (2012); the Education in Palliative and End-of-Life Care (EPEC) curriculum (Robinson et al., 2004); and ONCOTALK programs (Back et al., 2007) to teach these communication skills; however, there is little preparation or review for those already practicing. The majority of the research on communicating bad news has been focused primarily on medical oncology fellows.

Because research is largely physician-focused, there is a need for a wider examination of the role of the AP in initiating and having difficult conversations. The AP and physician should build a therapeutic relationship with the patient, laying the foundation for critical conversations. In fact, the AP often has the time and the one-on-one interaction opportunity that encourages growth of the therapeutic relationship required to initiate a difficult conversation with a patient.

The purpose of this article is to provide a framework for having a difficult conversation with an oncology patient and to clarify the role of the AP in the initiation of this critical communication.

## Communication Strategies

When an AP recognizes the potential for a difficult conversation, the first thing to do is set the stage for the conversation. The environment should be prepared by securing privacy, quieting the pager, reducing noise, gathering enough chairs, and sitting down. The AP can then draw on a number of different strategies, including ask-tell-ask, SPIKES, and NURSE.

The "ask-tell-ask" strategy (Table 1) allows the AP to check the patient’s expectations, deliver information, and then assess for understanding. The SPIKES tool (Table 2), a six-step protocol for giving bad news, is a well-recognized strategy involving setting the stage, delivering bad news, and concluding with a plan (Baile et al., 2000). The NURSE tool (Table 3) also allows the AP to address and respond to the emotional response of the patient (Campbell et al., 2010), which is critical when holding an emotionally laden conversation.

**Table 1:**
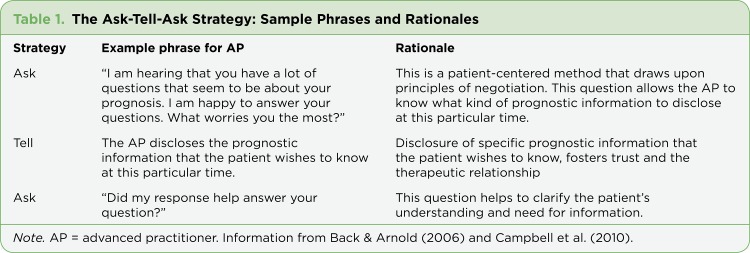
Table 1. The Ask-Tell-Ask Strategy:Sample Phrases and Rationales

**Table 2:**
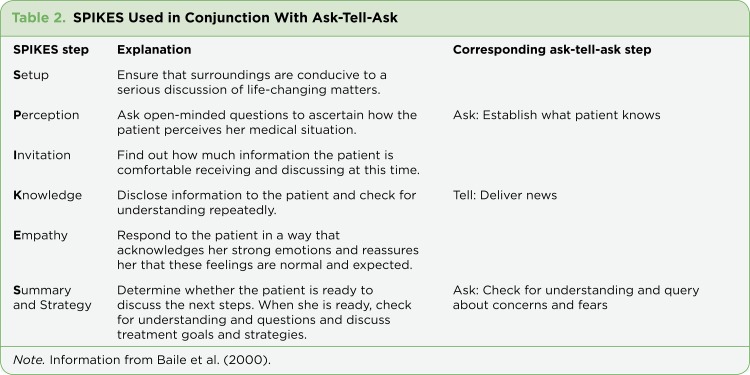
Table 2. SPIKES Used in Conjunction With Ask-Tell-Ask

**Table 3:**
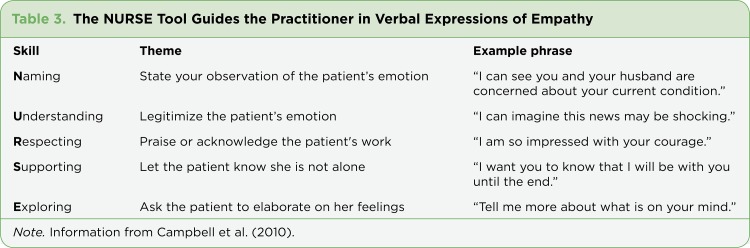
Table 3. The NURSE Tool Guides the Practitioner in Verbal Expressions of Empathy

Therapeutic silence can also be helpful in discerning or recognizing cues. Silence can be enriching, comforting, affirming, and safe. Silence can also be used to allow the patient and the clinician time for understanding and mutual respect (Back et al., 2009).

Regardless of the theoretical framework employed, the AP should begin the conversation focusing on how much the patient wants to know about prognosis and treatment options. Some patients want to know everything about their prognosis (Table 1). On the opposite end of the spectrum, there are patients and families who do not want information or are ambivalent about prognostic information. Back and Arnold (2006) reported that about 20% of patients do not want to discuss prognostic information or only want to hear good news. Elucidating and understanding the level of information the patient wants is critical in fostering trust between the patient and the AP (Back & Arnold, 2006). Acknowledging the patient’s emotional concern can be the single most useful tool available in dealing with patients who do not want to talk about prognosis (Back & Arnold, 2006). In one study, Hagerty et al. (2005) found that 98% of patients (N = 218) wanted their provider to be realistic, provide time for questions, and individualize their prognosis.

## Case Study

Mrs. S. is a 43-year-old female who presented to her primary care physician with a palpable mass in the upper quadrant of her left breast and tenderness in her axilla. She had a mammogram and ultrasound-guided biopsy, which demonstrated ER/PR/HER2-positive invasive ductal carcinoma (stage III; T3, N1, M0). Her physician discussed her cancer diagnosis and referred her to a medical oncologist. Mrs. S. comes to the medical oncologist’s office with her husband to discuss treatment options.

## INITIATING THE CONVERSATION

The AP has the opportunity to cultivate a therapeutic relationship with Mr. and Mrs. S. at the time of diagnosis. The AP can discern how much information the patient and family want. The ask-tell-ask strategy can be most revealing in this conversation. The AP is able to share a great deal of information about the disease, treatment options, percentages, prognosis, etc., but first asks the patient how much information she would like to have regarding her disease and therapy. The AP discovers that Mrs. S. wants to know about treatment options such as surgery, chemotherapy, and radiation. She states that she wants to live as long as possible to watch her two young girls (ages 3 and 5) grow up. She also reports that her family has a tradition in which the whole family goes to Disney World when the youngest daughter turns 5, and that going on this trip is a high priority.

Based on Mrs. S.’s response, the AP provides the appropriate information. Next, the practitioner goes to the second "ask" phase to clarify the patient’s understanding and to discern what other information she would like. Mr. and Mrs. S. state that while they are very overwhelmed with the diagnosis and would like some time to digest all of the new information, they are interested in starting treatment as soon as possible.

## PROGRESSION OF DISEASE

At 2 years post chemotherapy, radiation, and surgery, Mrs. S. is continuing with hormonal therapy. In January, she comes to the office with her husband for routine follow-up. She complains of new hip pain that is unrelieved by ibuprofen. The pain started approximately 3 weeks ago, after her last clinic visit. It is a constant, aching pain, which she rates a 7 on a 0–10 scale. She has never had pain like this before and now uses a cane to ambulate. She also reports a new cough that started approximately 2 months ago. CT and bone scan reveal numerous lung and bone metastases. Her children are now 5 and 7, and the family trip to Disney World is planned for August.

The AP and Mrs. S. are facing a transition point on the disease trajectory. The AP will break the bad news to Mrs. S. using the "ask-tell-ask" strategy, asking for permission to discuss the scan at this time. When Mrs. S. agrees to discuss the scan, the AP reveals that the scans show that the cancer has spread to the bone and the lungs and is the source of her hip pain. Next, the AP uses silence so that Mrs. S. and her husband can begin to absorb and process this new information.

Lastly, the AP asks the couple, "What does metastatic disease mean for you?" The way Mrs. S. answers this question can give the AP several layers of information. It sheds light on her emotional state. It also allows her to verbally express the meaning that she attaches to her disease progression. It can be a starting point for continuing the conversation about goals of treatment and palliative care.

Since the last visit in January, Mrs. S. has undergone radiation therapy to her hip with little relief. She also completed systemic chemotherapy, with progressive disease. She now returns for 6-month follow-up to discuss possible enrollment in a phase I clinical trial. She is more fatigued, resting most of the day. Her pain is unrelieved even after rotating opioids. She now has difficulty breathing due to recurrent malignant effusions, and a PleurX catheter has been placed. She also requires oxygen therapy.

Mrs. S. is worried about treatment options: starting a phase I clinical trial, receiving more systemic chemotherapy, or opting for no further treatment. She states, "Back in January, my husband and I discussed the possibility of going on the Disney trip earlier than planned. But we decided to keep it scheduled for August, hoping that I would feel better after treatment. Now I wish we had gone earlier. I’m already so tired and I want to be there for my family. I know my time is short and I’m scared. I don’t think I want any more treatment to prolong my life; I don’t like the quality of life I have now. I just want to spend time with my family and my girls."

## TRANSITION TO HOSPICE

Using the "ask-tell-ask" strategy, the AP discerns that Mrs. S. is afraid of dying. She does not feel that she is ready but understands that she will die soon; she is fearful for her family and children. The AP needs to reestablish the goals of care for Mrs. S. This strategy can be useful in deciding what is most important for Mrs. S. at this point in time. In reestablishing goals of care, using the "ask-tell-ask" strategy allows the AP to identify emotions and establishes that the conversation is about prognosis. Then the AP can determine what is most concerning at this time in order to elucidate how much information the couple wants. Once the current goals are identified—the most important of which is spending as much time with her family as possible—the AP can focus on helping Mrs. S. achieve this goal.

It is also important to establish additional goals of care regarding life-sustaining treatments. This is a good point to discuss a living will, durable power of attorney for health care, and code status, although Mrs. S. has been reluctant to discuss these issues in the past. Mrs. S. clearly states that she does not want life-sustaining treatment. The AP needs to ascertain what Mrs. S.’s definition of life-sustaining treatment is. Mrs. S. replies that she interprets this as meaning no more chemotherapy or anything that would prolong her life. The AP asks if they can discuss other life-prolonging treatments, such as CPR, ICU care, ventilators, and feeding tubes, while Mrs. S. is able to voice her decisions. When Mrs. S. agrees, she and the AP discuss these treatments and their effectiveness in how they relate to the goals of care and quality of life.

Hospice care can be introduced at this point as well. The AP explains hospice care and transition in care and the fact that hospice is focused on the quality of life and comfort of the patient and family. Mrs. S. learns that the hospice team will help with managing her fatigue and pain so she can be with her family. Hospice acknowledges and respects her goals of care, most important of which is to spend time with her family. The hospice team will work with Mrs. S to plan quality family activities. The AP reassures Mrs S. that she will stay connected to her and the hospice team, and that she is not going abandon her at this time of transition.

## Summary

Communication, a vital aspect of the patient/AP relationship, can include having difficult conversations with the patient to determine knowledge deficits and provide information in a safe and caring environment. But managing successful difficult conversations is a skill that needs to be taught and practiced—many APs are not formally instructed in this crucial skill. It is important for APs to seek out educational programs to strengthen communication skills. Finding a mentor who is proficient in having difficult conversations can be helpful in gaining this type of experience. There are several different models and strategies for having difficult conversations and breaking bad news. It is important for the AP to become comfortable using each approach and also to learn to choose the right strategy for the situation at hand.

Most of the research conducted thus far has been related to physicians. While it is reasonable to generalize this information to include APs, future research should include APs and their unique role in initiating and having difficult conversations.

Avoiding difficult discussions can limit the patient’s ability to pursue beneficial treatments and services (Campbell et al., 2010). Advanced practitioners need to deliver news, attend to the person, respond empathically, and address the patient’s emotions (Campbell et al., 2010). An AP well-skilled in having difficult discussions can help the patient and family to hope for the best but be prepared for the worst (Evans, Tulsky, Back, & Arnold, 2006).
